# Downregulation of PART1 Inhibits Proliferation and Differentiation of Hep3B Cells by Targeting hsa-miR-3529-3p/FOXC2 Axis

**DOI:** 10.1155/2021/7792223

**Published:** 2021-08-24

**Authors:** Zhicheng Weng, Jianyang Peng, Weida Wu, Chunsheng Zhang, Jianfeng Zhao, Hongbin Gao

**Affiliations:** ^1^Department of Intervention, Affiliated Hospital of Putian University, Putian, Fujian, China; ^2^Department of Hepatobiliary Surgery, Affiliated Hospital of Putian University, Putian, Fujian, China

## Abstract

**Background:**

Long noncoding RNAs (lncRNAs) are an important subtype of noncoding RNAs (ncRNAs) and microRNA sponges regulate protein-coding gene expression. The lncRNA prostate androgen-regulated transcript 1 (PART1) was implicated in the process of several cancer pathogeneses. However, studies on the regulation of PART1 expression and its mechanism in liver cancer are lacking.

**Methods:**

qRT-PCR and western blot were used to detect PART1 levels in liver cancer serums and cell lines. Cell proliferation, migration, and invasion were detected using CCK8 assays, cell clones, and transwell assays. Interaction between PART1 and miR-3529-3p and forkhead box protein C2 (FOXC2) was confirmed using dual-luciferase reporter assays.

**Results:**

We revealed that expression levels of PART1 and FOXC2 are significantly upregulated and the miR-3529-3p expression level significantly decreases in the serum while high expression level of PART1 is positively associated with tumour size, BCLC stage, and TNM stage. shRNA of PART1 can significantly reduce the ability of cell migration and invasion by regulating AKT signalling associated with the reduction of MMP-2 and MMP-9 protein expression. Dual-luciferase reporter assays showed that PART1 can sponge miR-3529-3p, which targets FOXC2 in liver cancer cells. The promoting or suppressing effect of PART1 for Hep3B cell proliferation, invasion, and migration is revised by miR-3529-3p mimics and inhibitors.

**Conclusion:**

Results showed that downregulation of PART1 can partially inhibit proliferation and differentiation by targeting hsa-miR-3529-3p/FOXC2 axis.

## 1. Introduction

Long noncoding RNAs (lncRNAs) are an important subtype of noncoding RNAs (ncRNAs) and are ubiquitous in organisms despite their lack of protein-encoding abilities [1]. LncRNAs are greater than 200 nucleotides as microRNA (miRNA) sponges regulating gene expression [2]. A previous study indicated that lncRNAs are an important section in competitive endogenous RNA [2, 3]. lncRNAs play key roles or pivotal functions in processes of tumour-related tumour formation, including proliferation, invasion, and metastasis [4–6]. The lncRNA prostate androgen-regulated transcript 1 (PART1) can regulate the proliferation and apoptosis of prostate cancer cells [7]. Moreover, lncRNA PART1 functions as a carcinogenic pyrene in non-small-cell lung cancer [8] and colorectal cancer [9]. The upregulation of lncRNA PART1 expression in the central nucleus pulposus tissue of patients with intervertebral disc degeneration can promote the intervertebral disc degeneration in nucleus pulposus cells by inhibiting the miR-93/MMP2 pathway [10]. The ability of lncRNA PART1 to suppress tumours by sponging miR-190a-3p suggested that lncRNA PART1 affects the function of miR-190a-3p by inactivating the AKT pathway [11]. However, the role of PART1 in liver cancer remains unverified.

According to the results of the target gene prediction software Diana Tools (https://carolina.imis.athena-innovation.gr), possible miRNA combinations for the selected lncRNA PART1 were chosen and showed the hsa-miR-3529-3p was the potential binding miRNA. Forkhead box protein C2 (FOXC2) was chosen as the target gene of potential binding miRNA miR-3529-3p by the TargetScanS tool (https://www.targetscan.org). The results indicated that the high expression of FOXC2 in hepatocellular carcinoma (HCC) is associated with malignant degree and poor prognosis, thereby suggesting that FOXC2 is an important prognostic factor in HCC [12]. Other studies have also indicated that the role of FOXC2 as a prognostic factor in HCC is related to EMT signalling pathway, including the E-cadherin, N-cadherin, vimentin, and ZEB1 proteins [12–14]. The downregulation of FOXC2 gene can inhibit HCC cell growth, migration, and invasion [13]. Moreover, FOXC2 is an important factor involved in tumour cell proliferation, differentiation, and invasion [15–18]. FOXC2 regulates the migration and invasion of extrahepatic cholangiocarcinoma by increasing the N-cadherin, Ang-2, and MMP-2 expression [16]. Previous results demonstrated that Akt-mediated activation of the MMP protein is involved in the FOXC2 function of promoting aggressive phenotypes [12, 16, 17]. However, determining whether FOXC2 participates in the liver cancer development and mediates the Akt signalling pathway as a target gene of miR-3529-3p requires further investigation.

Therefore, the present study aimed to investigate the potential role of lncRNA PART1 and its expression pattern in regulating the occurrence and development of liver cancer through miR-3529-3p by targeting the FOXC2-mediated Akt signalling pathway in Hep3B cells.

## 2. Materials and Methods

### 2.1. Blood Sample

Blood samples were collected from normal people and patients with liver cancer. The study was approved by the Ethical Committee of the Affiliated Hospital of Putian University. Informed written consent was obtained from participants of the study. Study subjects (*n* = 30 each) comprised normal people and patients with liver cancer. Blood samples were stored at −80°C for further analyses.

### 2.2. Cell Line and Cell Viability Assay

Hepatocyte (LO2) strain and hepatoma cell lines (HepG2, HuH7و and Hep3B) were purchased from Zolgene Biotechnology Co., Ltd (Fuzhou, China) and then cultured in a 5% CO_2_ incubator (SANYO, Osaka, Japan) at 37°C with DMEM/high-glucose medium (11965–092, Gibco^®^, NY) and 10% heat-inactivated fetal bovine serum (PAN biotech). The cell proliferation ability was measured using CCK-8 value and colony-forming assay. The CCK-8 value was determined with CCK-8 kits (CK04, Dojindo). Cells (1 × 10^5^ cells/mL) were adjusted into 24-well plates to incubate for 0, 24, 48, 72, and 96 h. CCK-8 (100 *μ*L) was added to each well plate. Absorbance values were recorded using a Biotek Synergy 2 plate reader (Winooski, VT) at 450 nm after incubating at 37°C for 1 h.

The colony-forming result was assayed via crystal violet method. Cells were inoculated in six-well plates, with 800 cells/well in each group. Inoculated cells were shaken and placed gently in the incubator for further culturing. Supernatant was discarded until the number of cells in most single clones in the well was greater than 50 and then washed once with PBS. Paraformaldehyde (4%, 1 mL) was added to each well and fixed at 4°C for 60 min. Crystal violet (0.1%, 1 mL) was subsequently placed into each well for 2 min. The results were recorded at a magnification of 100× (Olympus, CKX41).

### 2.3. Plasmid Construction and Mimics/Inhibitors/shRNA for Transfection

A psiCHECK^TM^-2 basic vector (Promega Corporation, USA) was used to clone wildtype (WT) PART1 3′-UTR. The full-length open reading frame of FOXC2 was cloned into pcDNA3.1 to generate expression vectors. The miR-3529-3p site-directed mutagenesis sequence was performed using the QuikChange™ Site-Directed Mutagenesis Kit (Stratagene, USA). PART1 WT, PART1 mutant-type, FOXC2 WT, and FOXC2 mutant-type plasmids were constructed. According to the PART1 gene information, shRNA sequences targeting the PART1 gene were designed and synthesised by Sangon Biotech (Shanghai, China). All mimics, inhibitors, and shRNA were designed and constructed by Sangon Biotech (Shanghai, China). The primer information is presented in Table 1. Mimics, inhibitors, or shRNAs were transfected into the cell with Lipofectamine 2000 (Invitrogen, L3000015) according to the manufacturer's protocol. The final concentration of mimics, inhibitors, and shRNAs was 20, 40, and 50 nM, respectively. Further experimentation was performed at least 48 h after cell transfection and transfection efficiencies were verified.

### 2.4. Luciferase Reporter Assays

LightSwitch luciferase assay reagents were obtained from Promega (Corporation, USA). The luciferase reporter plasmid and psiCHECK^TM^-2 vector expressing Renilla luciferase were transfected together with miR-3529-3p by overexpressing HEK293T for 24 h. Luciferase activity was measured using a dual-reporter assay system according to the manufacturer's instructions. The normalisation level was used in the Renilla luciferase.

### 2.5. Interaction Analysis between miR-3529-3p and PART1 or FOXC2

PART1 and miR-3529-3p or FOXC2 and miR-3529-3p were cotransfected into Hep3B cells and then inoculated into six-well plates 24 hours before transfection using logarithmic growth phase cells. Lipofectamine 2000 was used according to its operating procedure in the instruction manual. Gene and protein expression levels were detected via qPCR and WB methods, respectively, after 48 hours of transfection.

### 2.6. Migration and Invasion Ability Assay Using Transwell

Hep3B cells (1 × 10^5^ cells/mL) were suspended in 2 mL of serum-free medium. Hep3B cells (100 *µ*L) were added to the upper surface of the transwell chamber. The medium containing 20% FBS (600 *µ*L) was added to the lower surface of the transwell chamber and then cultured at 37°C for 24 h. Cells in the upper compartment were sampled with a cotton swab after culturing for 24 h. The medium in both chambers was removed, and 600 *μ*L of 4% paraformaldehyde was added to the lower chamber to fix cells for 30 min. Paraformaldehyde was removed, and cells were then sampled using a cotton swab with care to preserve membrane cells. Crystal violet (600 *μ*L, 0.1%, Sigma, Beijing, China) was added to the lower chamber for 15 min. Hep3B cells (1 × 10^5^ cells/mL) were cultured in the transwell chamber with extracellular matrix gel (Sigma, E1270) and diluted in ice with the serum-free medium at a ratio of 1 : 8 for transwell invasion assays. Cells were inoculated in the transwell chamber and cultured in 10% FBS medium for 24–48 h. Then, cells remaining in the top chamber were removed. Excess cells in the chamber and the residual Matrigel were removed with a cotton swab. Cells were fixed with paraformaldehyde and stained with 0.1% crystal violet (Sigma, Beijing, China) using the lower surface of the membrane cell after washing thrice with the PBS buffer. Fields of view at a magnification of 100× (Olympus, CKX41) were counted and expressed as the average number of cells per field of view.

### 2.7. qPCR Analysis

The total RNA was extracted from HepG2, HuH7, and Hep3B cells and the blood sample using the TRIzol reagent (Invitrogen, USA). The miRNA was isolated with a miRNeasy Mini Kit (Qiagen, Germany), and reverse transcription and qPCR analysis were performed according to the method used in a previous study [19]. The qPCR SYBR® Green Master Mix (A6001, Promega, USA) was used and combined with 2.0 *μ*L of reverse transcription product, 7.0 *μ*L of nuclease-free water, and 0.5 *μ*L of forward and reverse primers in real-time PCR. The mRNA expression was subsequently detected using the ABI 7500 PCR system (ABI, USA). The result was calculated via the 2^−△△Ct^ method [20]. The following qPCR conditions were applied: 95 °C for 5 min and 40 cycles at 95 °C for 10 s, 60 °C for 30 s, and 72 °C for 30 s. Primers were designed using Primer 5.0 software and then synthesised by Sangon Biotech (Shanghai, China). The miR-3529-3p primer sequence is GCGCGAACAACAAAATCACTAGT (forward) and AGTGCAGGGTCCGAGGTATT (reverse) with an RT primer of GTCGTATCCAGTGCAGGGTCCGAGGTATTCGCACTGGATACGACTGGAAG. The U6 primer sequence is CTCGCTTCGGCAGCACATATACT (forward) and ACGCTTCACGAATTTGCGTGTC (reverse) with an RT primer of AAAATATGGAACGCTTCACGAATTTG. The lncRNA PART1 primer is CTTCTCGTACGCTGGGCTAT (forward) and TTGTTCCAGTGCAGCCCTTT (reverse). The FOXC2 primer is CGGCGGCGCTTCAAAAAGA (forward) and CGCTCTTGATCACCACCTTCTTC (reverse). The GAPDH primer is ATGGGGAAGGTGAAGGTCG (forward) and TTACTCCTTGGAGGCCATGTG (reverse).

### 2.8. Protein Expression Analysis

Protein samples were extracted from Hep3B cells with the RIPA buffer, including PMSF, on ice for 20 min at 4°C. The supernatant solution (200 *μ*L) was collected in an EP tube after centrifuging at 12 000 rpm for 20 min. The BCA Protein Assay Kit PC0020-500 (Solaibao Technology Co. Ltd, Beijing, China) was used to detect the protein concentration. Proteins were separated using SDS-polyacrylamide gel step according to the previous description [20, 21] and the separated protein was blocked. The blocking protein was washed with TBS buffer, including 0.5% Tween-20 (Zolgene Biotechnology Co., Ltd, Fuzhou, China), and incubated overnight at 4°C with anti-FOXC2 MMP-2, MMP-9, and *β*-actin (1 : 1500, Abcam, Beijing, China). Primary antibodies were washed with Tris-buffered saline containing 0.1% Tween-20 after incubation. Samples were incubated with an antimouse HRP-conjugated secondary antibody (1 : 5000 dilution) for 2 h. The signal was detected using chemiluminescence with Thermo ECL Substrate (Bio-Rad). Membranes incubated with *β*-actin (1 : 1000, Abcam, Beijing, China) were used as loading controls. The Versa DocTM imaging system (Peiqing Technology Co. Ltd, Shanghai, China) was utilised to collect the results, which were analyzed with Image J software.

### 2.9. Statistical Analysis

SPSS 22.0 statistical software was applied to conduct statistical analyses. Data are expressed as mean ± SD. Student's *t*-test or one-way ANOVA was performed for all experiments with more than two groups. *P* < 0.05 was considered statistically significant.

## 3. Results

### 3.1. Effect of lncRNA PART1, miR-3529-3p, and FOXC2 Expression on Liver Cancer

**Figure 1:**
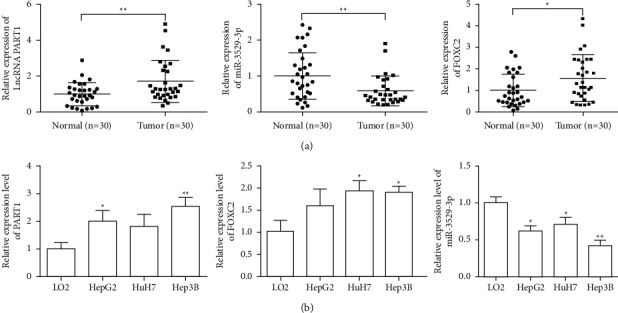
Effect of lncRNA PART1, miR-3529-3p, and FOXC2 expression on liver cancer. (a) The relative expression of PART1, miR-3529-3p, and FOXC2 in serum of patients and normal people was analyzed by qRT-PCR. (b) The relative expression of PART1, miR-3529-3p, and FOXC2 in cell lines was analyzed by qRT-PCR. All of the data are presented as the mean ± SD. Compared with control group, ^*∗*^*P* < 0.05; ^*∗∗*^*P* < 0.01.

As shown in Figure 1(a), expression levels of PART1 and FOXC2 were significantly upregulated in the serum of liver cancer patients but that of miR-3529-3p significantly decreased (*P* < 0.05 or *P* < 0.01) compared with that of the normal group. As shown in Figure 1(b), expression levels of PART1 and FOXC2 were significantly upregulated but that of miR-3529-3p was significantly downregulated (*P* < 0.05 or *P* < 0.01) in HepG2, HuH7, and Hep3B cell lines compared with that of normal liver cells (LO2). Hep3B cells clearly reflect these results. Therefore, we selected Hep3B cells for subsequent experiments. The clinical results showed that PART1 is significantly overexpressed in liver cancer (Table 1), thereby indicating that PART1 is unrelated to gender, age, and metastasis of liver cancer but related to tumour size, TNM stage, and BCLC stage. Hence, PART1 may be a potential tumour marker for the occurrence and development of liver cancer (Table 1).

### 3.2. Effects of lncRNA PART1 on the Biological Function of Hepatoma Cells

**Figure 2:**
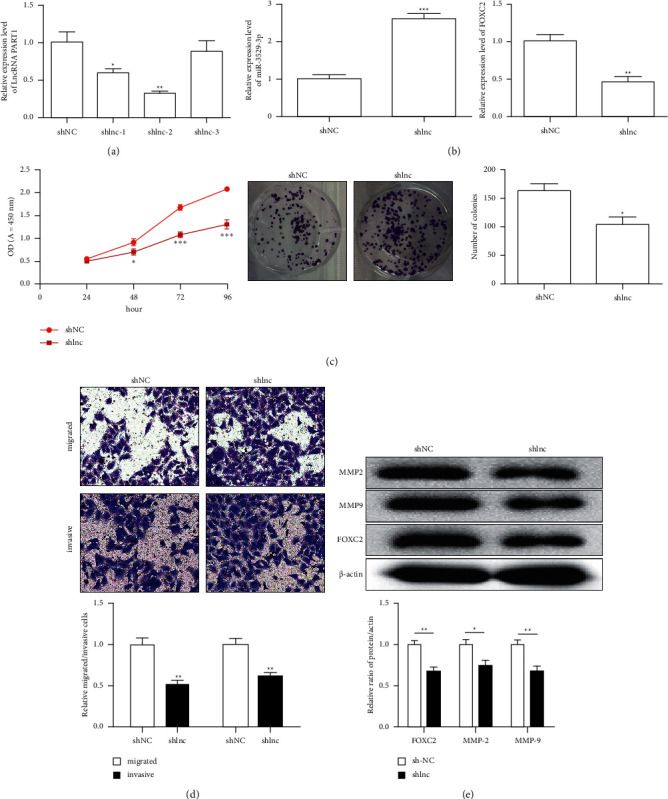
Effects of lncRNA PART1 on the biological function of hepatoma cells. (a) Transfection efficiency of shlnc; (b) the expression level of FOXC2 and miR-3529-3p in Hep3B cells transfected with the shlnc-2; (c) the cell viability of in Hep3B cells; (d) the ability of cell migration and invasion; (e) FOXC2, MMP-2, and MMP-9 protein expression in Hep3B cells transfected with shlnc-2. All of the data are expressed as the mean ± SD. Compared with the control group, ^*∗*^*P* < 0.05; ^*∗∗*^*P* < 0.01; ^*∗∗∗*^*P* < 0.001.

ShRNA was transfected to determine whether lncRNA PART1 can regulate the biological function of hepatoma cells and establish Hep3B cells stably. The shlnc-2 is chosen on the basis of its optimum effect (Figure 2(a)). Compared with the shNC group, the expression level of FOXC2 was significantly downregulated and that of miR-3529-3p was significantly upregulated in the shlnc group after the transfection of shlnc-2 (*P* < 0.01 or *P* < 0.001, Figure 2(b)). Cell viability, including the CCK and clone formation results (*P* < 0.05, *P* < 0.01 or *P* < 0.001, Figure 2(c)), was significantly lower in the shlnc group than that in the shNC group. The ability of cell migration and invasion was significantly reduced with the shlnc transfection via the transwell test (*P* < 0.01, Figure 2(d)). To address the mechanism of how PART1 affects the Hep3B cell function by the regulating the AKT signalling caused by FOXC2 expression, the western blot results revealed that the FOXC2 protein expression is significantly downregulated in the shlnc group compared with that in the shNC group. This significant downregulation can inhibit the Akt signalling pathway. Protein expression levels of MMP-2 and MMP-9 were significantly downregulated (*P* < 0.05 or *P* < 0.01, Figure 2(e)).

### 3.3. Effects of miR-3529-3p on Hepatoma Cells

**Figure 3:**
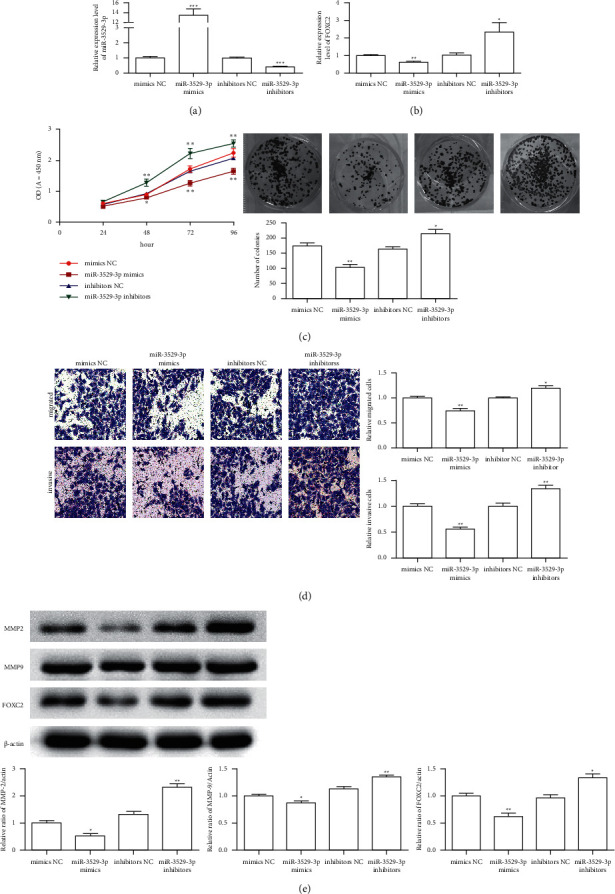
Effects of miR-3529-3p on hepatoma cells. (a) The expression level of miR-3529-3p; (b) the expression level of FOXC2; (c) the cell viability of Hep3B cells was analyzed by CCK-8 analysis and the clone formation; (d) the transwell invasion assays of Hep3B cells transfected with the miR-3529-3p mimic and the miR-3529-3p inhibitor; (e) FOXC2, MMP-2, and MMP-9 protein expression in Hep3B cells. All protein samples were isolated in Hep3B cells after transfection with miR-3529-3p mimic and the miR-3529-3p inhibitor. All of the data are expressed as the mean ± SD. Compared with control group, ^*∗*^*P* < 0.05, ^*∗∗*^*P* < 0.01, ^*∗∗∗*^*P* < 0.001.

Mimics and inhibitors were transfected into Hep3B cells to determine whether miR-3529-3p can regulate the biological function of hepatoma cells. The results in Figure 3(a) showed that the expression level of miR-3529-3p in the mimic group is significantly upregulated compared with that of the mimic NC group. The miR-3529-3p expression level in the inhibitor group was significantly downregulated compared with that of the inhibitor NC group (*P* < 0.001). The expression level of FOXC2 was significantly downregulated after transfection of miR-574-5p mimics but significantly upregulated in the miR-3529-3p inhibitor group compared with that in the NC group (*P* < 0.01 or *P* < 0.05Figure 3(b)). Cell viability, including the CCK result and clone formation result (*P* < 0.05, *P* < 0.01 or *P* < 0.001, Figure 3(c)), significantly decreased in the miR-3529-3p mimic group but increased when transfected with miR-3529-3p inhibitor after incubation for 48 (*P* < 0.05), 72 (*P* < 0.01), and 96 (*P* < 0.01) *h*. The transwell test results indicated that the miR-3529-3p mimic transfection significantly reduces the ability of cell migration and invasion but increases with the miR-3529-3p inhibitor transfection (*P* < 0.01, Figure 3(d)). The western blot results showed that FOXC2, MMP-2, and MMP-9 protein expression levels were significantly downregulated in the miR-3529-3p mimic group but upregulated with the miR-3529-3p inhibitor transfection (*P* < 0.05 or *P* < 0.01, Figure 3(e)) compared with those of the mimic NC group. These results indicated that miR-3529-3p affects the Hep3B cell function by regulating the AKT signalling caused by the FOXC2 expression.

### 3.4. Effect of PART1-hsa-miR-3529-3p on Regulating the Occurrence and Development of Liver Cancer

**Figure 4:**
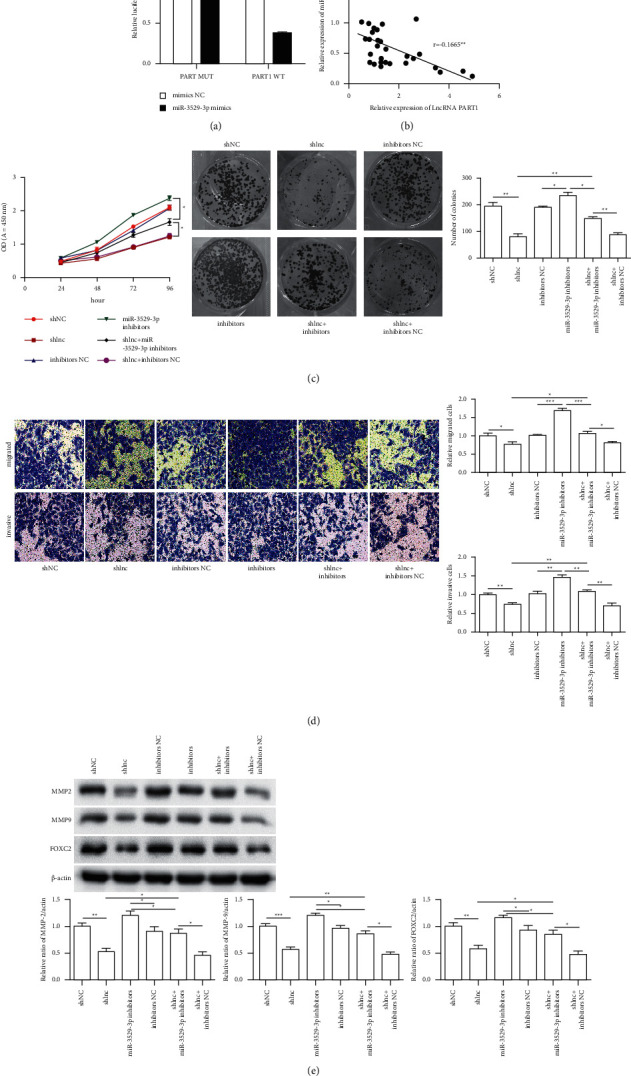
Effect of PAPT1-hsa-miR-3529-3p on regulating the occurrence and development of liver cancer. (a) PAPT1 is a target of miR-3529-3p. Left: schematic representation of the miR-3529-3p site in the PAPT1 3′-UTR. Right: the 3′-UTR reporter assay was carried out in Hep3B transfected with miR-3529-3p mimic or mimic NC. The MUT or WT reporter plasmids were transfected with Lipo-2000. Luciferase assays were performed 48 h after transfection. Firefly luciferase activity was standardized to a Renilla luciferase control. (b) PAPT1 expression was negatively correlated with hsa-miR-3529-3p expression in liver cancer serums. (c) The cell viability of Hep3B cells including the CCK analysis and clone formation; (d) the transwell invasion assays of Hep3B cells transfected with the shlnc and the miR-3529-3p inhibitor; (e) FOXC2, MMP-2, and MMP-9 protein expression in Hep3B cells. All protein samples were isolated in Hep3B cells after transfection with shlnc and the miR-3529-3p inhibitor. Compared with control group, ^*∗*^*P* < 0.05; ^*∗∗*^*P* < 0.01; ^*∗∗∗*^*P* < 0.001.

The luciferase reporter assay results indicated that the luciferase activity of the PART1 3′-UTR in the overexpression of miR-3529-3p cells is significantly lower than that in the mimic control-transfected cells (Figure 4(a)) and miR-3529-3p shows a negative relation with PART1 in the expression (Figure 4(b)). This finding suggested that miR-3529-3p reduces the PART1 expression by binding to target sites. Cell viability significantly increased in the shlnc + miR-3529-3p inhibitor group compared with that of shlnc and shlnc + inhibitor NC groups. However, viability, including the CCK result and clone formation result (*P* < 0.05, *P* < 0.01 or *P* < 0.001, Figure 4(c)), significantly decreased in the shlnc + inhibitor group compared with that of the miR-3529-3p inhibitor group. Cell migration and invasion ability significantly increased in the shlnc + miR-3529-3p inhibitor group compared with that of shlnc and shlnc + inhibitor NC groups. However, viability significantly decreased in the shlnc + inhibitor group compared with that of the miR-3529-3p inhibitor group (*P* < 0.05, *P* < 0.01 or *P* < 0.001, Figure 4(d)). The western blot results revealed that FOXC2, MMP-2, and MMP-9 protein expression levels were significantly upregulated in the shlnc + miR-3529-3p inhibitor group compared with those of shlnc and shlnc + miR-3529-3p inhibitor NC groups but significantly decrease in the shlnc + miR-3529-3p inhibitor group compared with those of the miR-3529-3p inhibitor group (*P* < 0.05 or *P* < 0.01, Figure 4(e)). These results suggested that the reduction of PART1 and miR-3529-3p expression levels can increase FOXC2 and Akt signalling protein expression levels in Hep3B cells.

### 3.5. Effect of the hsa-miR-3529-3p/FOXC2 Axis on Regulating the Occurrence and Development of Liver Cancer

**Figure 5:**
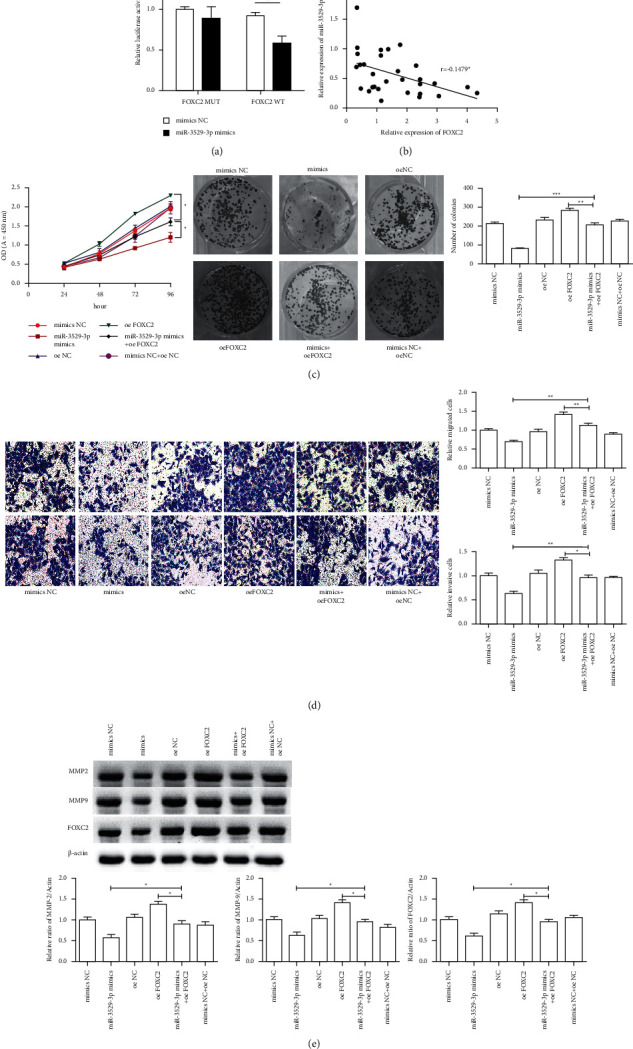
Effect of hsa-miR-3529-3p/FOXC2 axis on regulating the occurrence and development of liver cancer. (a) FOXC2 is a target of miR-3529-3p. Left: Schematic representation of the miR-3529-3p site in the FOXC2 3′-UTR. Right: The 3′-UTR reporter assay was carried out in Hep3B transfected with miR-3529-3p mimic or mimic NC. The MUT or WT reporter plasmids were transfected with Lipo-2000. Luciferase assays were performed 48 h after transfection. Firefly luciferase activity was standardized to a Renilla luciferase control. (b) hsa-miR-3529-3p expression was negatively correlated with FOXC2 expression in liver cancer serums. (c) The cell viability of Hep3B cells including the CCK analysis and clone formation. (d) The transwell invasion assays of Hep3B cells transfected with the miR-3529-3p mimic and the overexpression of FOXC2; (e) FOXC2, MMP-2, and MMP-9 protein expression in Hep3B cells. All protein samples were isolated in Hep3B cells after transfection with the miR-3529-3p mimic and the overexpression of FOXC2. Compared with control group, ^*∗*^*P* < 0.05; ^*∗∗*^*P* < 0.01; ^*∗∗∗*^*P* < 0.001.

Figure 5(a) shows that the luciferase activity of FOXC2 3′-UTR in the overexpression of the miR-3529-3p cell significantly reduces compared with that of mimic control-transfected cells (Figure 5(a)) and FOXC2 shows a negative relation with miR-3529-3p in expression (Figure 5(b)). Hence, miR-3529-3p reduces the FOXC2 expression by binding to target sites. Cell viability, including CCK result and clone formation result (*P* < 0.05, Figure 5(c)), in the miR-3529-3p mimic + oeFOXC2 group was higher than that in the miR-3529-3p mimic group but significantly decreases compared with that of the oeFOXC2 group. Figure 5(c) presents that cell migration and invasion ability significantly increase in the miR-3529-3p mimic + oeFOXC2 group compared with those in the miR-3529-3p mimic group but significantly decrease compared with those of the oeFOXC2 group (*P* < 0.05, Figure 5(d)). The western blot results revealed that FOXC2, MMP-2, and MMP-9 protein expression levels were significantly upregulated in the miR-3529-3p mimic + oeFOXC2 group compared with those in the miR-3529-3p mimic group but significantly decrease compared with those of the oeFOXC2 group (*P* < 0.05, Figure 5(e)). These results showed that miR-3529-3p targets FOXC2 to regulate AKT signalling.

## 4. Discussion

lncRNAs are important noncoding RNAs (ncRNAs) that function as microRNA regulators that control the protein-coding gene expression [1, 2]. A previous study indicated that lncRNAs are important regulators that play pivotal functions in controlling tumour-related tumour formation, proliferation, cell invasion, and tumour metastasis [4–6]. PART1 regulates prostate cancer cell proliferation and apoptosis [7]. The lncRNA PART1 in the serum of liver cancer patients and cell lines were significantly upregulated in the present study. Notably, the lncRNA PART1 expression is clear in Hep3B cells. These results were similar to those of previous studies, which showed that lncRNA PART1 is a carcinogenic pyrene in non-small-cell lung cancer cell [8] and colorectal cancer [9]. A recent study revealed that lncRNA PART1 can suppress tumours by regulating miR-190a-3p and subsequently inactivating the AKT pathway [11]. Our results also showed that shRNA of lncRNA PART1 transfection significantly downregulates FOXC2 gene and protein expression levels as well as protein expression levels of MMP-2 and MMP-9 associated with the AKT pathway. The upregulated expression of lncRNA PART1 in the central nucleus pulposus tissue of patients can also promote the intervertebral disc degeneration in the nucleus pulposus cell by inhibiting the miR-93/MMP2 pathway [10].

The miR-3529-3p expression level in this study was significantly upregulated with shlnc-2 transfection, thereby suggesting that lncRNA PART1 is involved in regulating the miR-3529-3p expression. The biological role of miR-3529-3p in the biological function of hepatoma cells was investigated, and mimics and inhibitors were transfected into Hep3B cells. The transfection of miR-3529-3p inhibitors significantly reduced migration and invasion by inhibiting miR-3529-3p expression levels. This finding indicated that the miR-3529-3p expression exerts a strong positive correlation with tumour development. The transwell test results showed that miR-3529-3p is an important regulator because the miR-3529-3p mimic transfection significantly reduces cell migration and invasion but increases with the miR-3529-3p inhibitor transfection. The results of this study indicated that expression levels of FOXC2, MMP-2, and MMP-9 proteins were significantly downregulated in the miR-3529-3p mimic group.

Previous results indicated that the FOX gene is a biomarker and an important tumour suppressor gene [22–25] because it inhibits the growth, invasion, and migration of cancer cells [26, 27]. Li et al. [28] indicated that the FOX protein is essential for cancer complex and can regulate breast cancer development and metastasis by regulating cell differentiation. Sun et al. [29] showed that FOX can inhibit the liver cancer cell proliferation. Our results suggested that miR-3529-3p can bind target sites and reduce the PART1 expression. Cotransfection of PART1 and miR-3529-3p in Hep3B demonstrated that FOXC2, MMP-2, and MMP-9 protein expression levels were significantly upregulated in the inhibition of lncRNA PART1 and miR-3529-3p. Hence, liver cancer can be inhibited by suppressing lncRNA PART1 and miR-3529-3p expression on the basis of the blocking effect of FOXC2 and AKT protein expression.

Previous studies indicated that the MMP pathway can activate caspase family members and lead to a significant decrease in the ability of cell migration and invasion [30]. MMP2 and MMP9 belong to the metzincin superfamily and contribute to the pathogenesis of cancer cell invasion [31]. The MMP-2 expression increased in patients with tumours [32, 33]. MMP2 and MMP9 expression can be upregulated by suppressing the lncRNA PART1 and miR-3529-3p expression. Notably, shRNA and the inhibitor of PART1 and miR-3529-3p cotransfected in Hep3B cells can regulate the cell migration and invasion associated with the significant downregulation of FOXC2, MM2, and MM9 protein expression levels. A similar phenomenon was observed in XBP1 overexpressed melanoma cells, wherein the increasing expression of XBP1 promotes melanoma cell proliferation by activating the AKT pathway associated with MMP2 and MMP9 [34]. Moreover, the activation of Akt can protect against apoptosis induced by stimuli [35].

Overall, these data suggested that the downregulation of PART1 can inhibit the proliferation and differentiation of Hep3B cells by targeting the hsa-miR-3529-3p/FOXC2 axis and thus hinder the occurrence and development of liver cancer.

## Figures and Tables

**Table 1:** Clinical information analysis of the significance of PART1 expression in liver cancer.

Clinicopathological parameter	PART1 expression				
			Lower expression (%)	High expression (%)	*P* value
Gender	Male	24	12	12	1.000
	Female	6	3	3	

Age	≤65	19	8	11	0.2557
	>65	11	7	4	

Tumour size	<5 cm	15	11	4	0.0106^*∗*^
	≥5 cm	15	4	11	

Tumour metastasis	+	12	5	7	0.4561
	−	18	10	8	

BCLC stage	B	14	4	10	0.0281^*∗*^
	C	16	11	5	

TNM stage	I–II	14	4	10	0.0281^*∗*^
	III–IV	16	11	5	

*Note.* Compared with control group, ^*∗*^*P* < 0.05; ^*∗∗*^*P* < 0.01.

## Data Availability

All of the data are provided in the manuscript.
